# Distribution and Drug Resistance of Bacterial Infection in Hospitalized Patients at the Respiratory Department before and after the COVID-19 Pandemic in Guangzhou, China

**DOI:** 10.3390/microorganisms11102542

**Published:** 2023-10-12

**Authors:** Ling Hao, Xiao Yang, Huiling Chen, Shuquan Wei, Banglao Xu, Ziwen Zhao

**Affiliations:** 1 Department of Pulmonary and Critical Care Medicine, Guangzhou First People’s Hospital, School of Medicine, South China University of Technology, Guangzhou 510180, China; eyhaoling@scut.edu.cn (L.H.); eyweishuquan@scut.edu.cn (S.W.); 2 Department of Laboratory Medicine, Guangzhou First People’s Hospital, School of Medicine, South China University of Technology, Guangzhou 510180, China; eyyangxiao@scut.edu.cn (X.Y.); huiling1515@163.com (H.C.)

**Keywords:** COVID-19, bacterial infection, respiratory department, gram-negative bacteria, antimicrobial resistance, carbapenem-resistant bacteria

## Abstract

Since COVID-19 might have a lasting impact on global public health, it is crucial to analyze its effect on drug-resistant bacterial infections in the respiratory system for the prevention and control of hospital infections. This work aimed to investigate the impact of the COVID-19 outbreak on the clinical distribution and antibiotic resistance of bacterial infection among hospitalized patients in the respiratory unit in order to establish strategies to control antibiotic-resistant infections. Electronic clinical data registry records from 2018 to 2022 were retrospectively analyzed. A total of 36,829 clinical specimens, including sputum, bronchoalveolar lavage fluid, blood, and urine, were collected from 16,073 patients admitted to the Guangzhou First People’s Hospital from January 2018 to December 2022. Among them, 2209 samples were culture-positive. The bacterial isolation rates of different types of samples showed a similar trend from 2019 to 2022, with an increase in 2020 and 2022 and a decrease in 2021. Different bacterial species were separated from different types of samples. The most reported pathogens were identified in sputum samples. Gram-positive isolates were prevalent in urine samples, while Gram-negative bacilli were the predominant pathogenic bacteria isolated from respiratory tract and blood samples. *Pseudomonas aeruginosa* (*P. aeruginosa*), *Acinetobacter baumannii* (*A. baumannii*) complex, and *Klebsiella pneumoniae* (*K. pneumoniae*) were the most abundant Gram-negative bacteria in sputum samples, of which *A. baumannii* complex had the highest resistance to all tested antibiotics except colistin. Notably, there has been a substantial prevalence of carbapenem-resistant *P. aeruginosa*, *A. baumannii*, and *K. pneumoniae* in the past five years. This alarming situation calls for greater attention and precaution with prescribed antibiotics to limit the generation and spread of new multidrug-resistant bacteria and improve therapeutic management.

## 1. Introduction

The coronavirus disease 2019 (COVID-19) pandemic, caused by severe acute respiratory syndrome coronavirus 2 (SARS-CoV-2), has become an unprecedented public health crisis in the 21st century with over 769 million identified cases and over 6.9 million deaths reported [1]. The outbreak of the COVID-19 pandemic has changed the lifestyles of human beings and may have an inadvertent effect on the prevalence and resistance patterns of other respiratory pathogens [2].

Several reports have described an increase in the number of cases infected by antimicrobial-resistant pathogens during the COVID-19 pandemic [3,4]. A retrospective study showed that the incidence of carbapenem-resistant Enterobacteriaceae colonization increased more than seven-fold in 2020 compared to that in 2019 [3]. Similarly, an increase in the incidence of ampicillin/sulbactam, imipenem and levofloxacin resistance of *Acinetobacter baumannii* (*A. baumannii*) complex isolates was observed in the National Taiwan University Hospital during January–June 2020 compared with January–June 2019 [4]. The underlying reason for high antimicrobial resistance might be the unnecessary use of antibiotics during the COVID-19 pandemic. Studies have shown that more than half of COVID-19 patients had administered antimicrobials, and this number was much higher in patients with severe or critical disease [5,6,7]. A recent meta-analysis published in 2023 suggested that antimicrobial resistance was highly common in patients with COVID-19 and bacterial infections, with a prevalence rate of 60.8% [8]. In addition, during the peak of the epidemic, overcrowding in hospitals, a higher proportion of patients in intensive care units (ICUs), and fatigue among healthcare workers might exacerbate the spread of antibiotic resistance [9,10,11]. On the other hand, some studies, however, have demonstrated a decrease in antimicrobial resistance during the COVID-19 pandemic [12,13]. The reasons may be mainly related to the adoption of stringent infection control measures and the increased awareness of self-protection and hand hygiene.

Gram-negative bacteria such as *Escherichia coli* (*E. coli*), *Klebsiella pneumoniae* (*K. pneumoniae*), *Pseudomonas* species, and *Acinetobacter* species are common opportunistic human pathogens and play an important role in drug resistance dissemination in healthcare settings [14]. Empirical antibiotic treatment is commonly used for some respiratory diseases, such as lower respiratory tract infections, and drug-resistant bacteria are commonly detected in respiratory departments [15]. Since COVID-19 might have a lasting impact on global public health, it is crucial to analyze its effect on infections caused by drug-resistant bacteria in the respiratory system for the prevention and control of hospital infections. This was a retrospective study conducted at Guangzhou First People’s Hospital, a university-affiliated and tertiary hospital with 2970 beds. We collected the information and samples from hospitalized patients admitted to the Department of Pulmonary and Critical Care Medicine (PCCM) between January 2018 and December 2022 to investigate the changes in bacterial infections and associated antibiotic resistance profiles of the inpatients before and after the COVID-19 pandemic.

## 2. Results

### 2.1. Demographic and Clinical Characteristics of Inpatients

This work enrolled 16,073 patients who were admitted to the PCCM department of Guangzhou First People’s Hospital, Guangdong, China, from 2018 to 2022. As shown in Table 1, the number of male patients per year was nearly twice as many as that of female patients. More than half of patients were aged 65 years or above. In relation to co-morbidities, the proportion of patients suffering from hypertension, diabetes, chronic obstructive pulmonary disease (COPD) and asthma is 42.40%, 18.94%, 22.37%, and 4.25%, respectively.

### 2.2. Bacterial Detection from 2018 to 2022

**Figure 1:**
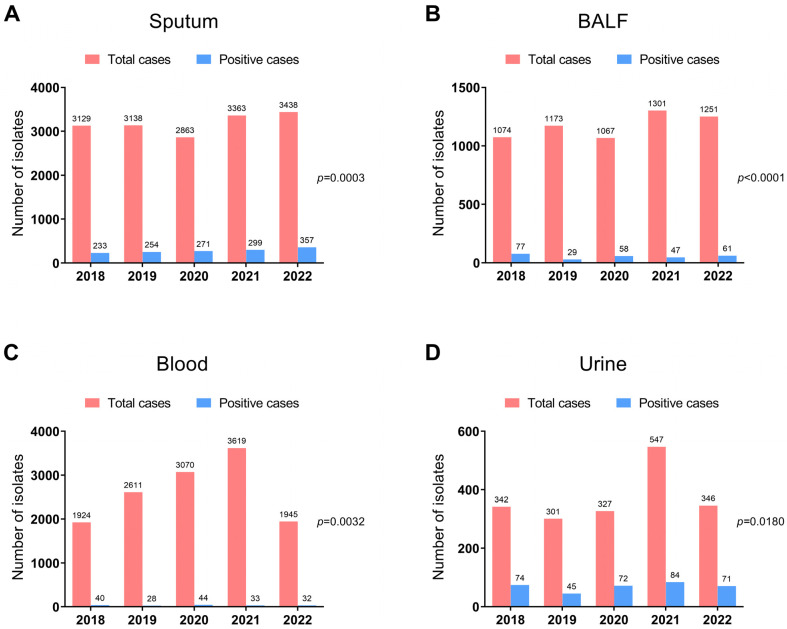
The total numbers and positive numbers of bacterial isolates identified in sputum (**A**), BALF (**B**), blood (**C**), and urine (**D**) of hospitalized patients in the PCCM department from 2018 to 2022. Statistical significance of the bacterial detection among different years was calculated by multiple independent sample contingency table χ^2^ (and Fisher’s exact) test.

A total of 36,829 clinical specimens, including sputum, bronchoalveolar lavage fluid (BALF), blood, and urine, were collected. Among them, bacterial pathogens were identified in 2209 samples, and these specimens were then designated as positive samples. We first compared the total numbers and the positive numbers of different types of samples to analyze the impact of the COVID-19 pandemic on the pathogen prevalence of respiratory inpatients in our hospital from 2018 to 2022. During the sample separation process, the positive numbers with the reported pathogen from sputum were the highest, whereas blood samples had the lowest positive counts. Compared to 2019, the total number of sputum and BALF cultures decreased, while the number of positive cultures increased in 2020, the first year of the COVID-19 pandemic (Figure 1A,B). In addition, a significant decrease was found in the total number of blood and urine samples in 2022 when compared to 2021 (Figure 1C,D).

**Figure 2:**
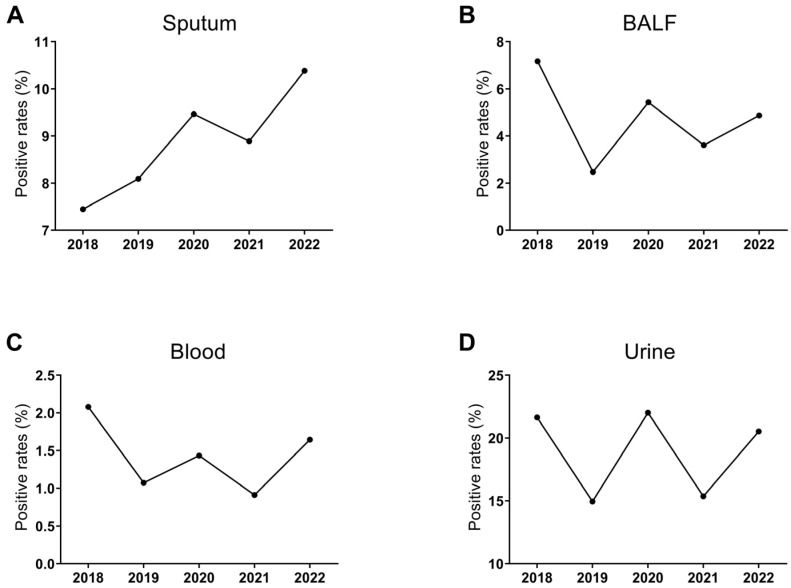
The positive rates of bacterial isolates identified in sputum (**A**), BALF (**B**), blood (**C**), and urine (**D**) of respiratory inpatients from 2018 to 2022.

The positive rates of the four specimens showed a similar trend from 2019 to 2022, with an increase in 2020 and 2022 and a decrease in 2021 (Figure 2). Notably, the positive rates of sputum cultures showed an increasing trend year by year except for 2021 (*p* <0.0001) (Figure 2A). In addition, the highest separation rates were found in urine (14.95~22.02%), while the positive rates of blood cultures were the lowest (0.91~2.08%) (Figure 2A–D).

### 2.3. Identified Bacteria from Tested Specimens

**Figure 3:**
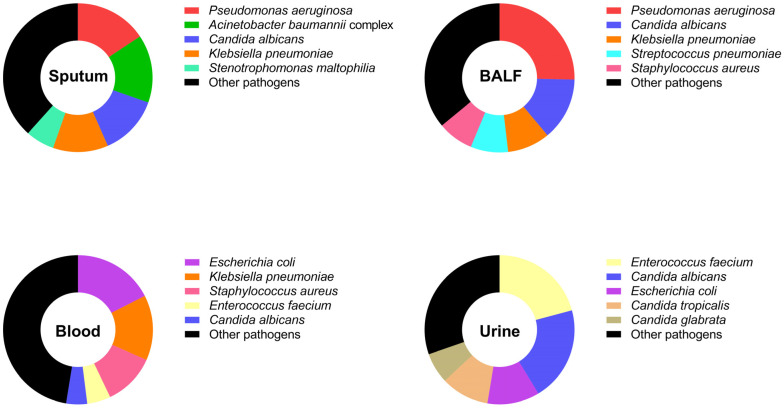
The total percentage of identified bacterial species isolated from different sample types (sputum, BALF, blood, and urine) of respiratory inpatients from 2018 to 2022.

The prevalence of detected Gram-positive and Gram-negative bacterial species in different types of specimens collected from patients in the PCCM department between 2018 and 2022 was investigated (Figure 3). In total, *P. aeruginosa* showed the highest prevalence in sputum and BALF samples, with a rate of 15.63% and 25.37%, respectively. *E. coli* was mainly identified in blood samples (17.51%), and *Enterococcus faecium* was linked to urine samples (20.81%). *K. pneumoniae* was mainly detected in sputum, BALF, and blood samples, accounting for 9.19~14.12% of identified bacterial species. In addition, *Staphylococcus aureus* was related to BALF (7.72%) and blood samples (11.30%), while *A. baumannii* complex was mainly associated with sputum samples (14.78%). In particular, we found that the proportion of a fungus, *Candida albicans*, in the tested specimens was relatively high during the study period (2018–2022), which was calculated as 13.01% in sputum samples, 13.60% in BALF samples, and 20.52% in urine samples. The details of pathogens identified in different types of samples are shown in Tables S1–S4.

The pathogen distributions with positive culture results before and after the COVID-19 pandemic were further analyzed. Among the positive sputum cultures, the most common pathogens included *P. aeruginosa*, *A. baumannii* complex, *Candida albicans*, *K. pneumoniae*, *Stenotrophomonas maltophilia*, *Haemophilus influenzae*, *Streptococcus pneumoniae*, and *Staphylococcus aureus* (Table 2), which accounted for over 75% of the total detected pathogens. Among the most common pathogens, a significant decrease in infections with *P. aeruginosa* was observed in 2020 (*p* = 0.0119). In addition, the positive rates of *Candida albicans* significantly decreased in 2019 (*p* = 0.0125) while remaining stable in the following years. *K. pneumoniae* increased (*p* = 0.0108), while *Streptococcus pneumoniae* decreased (*p* = 0.0303) from 2018 to 2020. Both bacteria kept a relatively stable state from 2020 to 2022. Moreover, *Stenotrophomonas maltophilia* significantly increased (*p* = 0.0401), while *Haemophilus influenzae* infections decreased (*p* < 0.0001) in 2020, and both remained stable from 2020 to 2022.

### 2.4. Antimicrobial Resistance Profiles of Detected Bacterial Isolates

**Figure 4:**
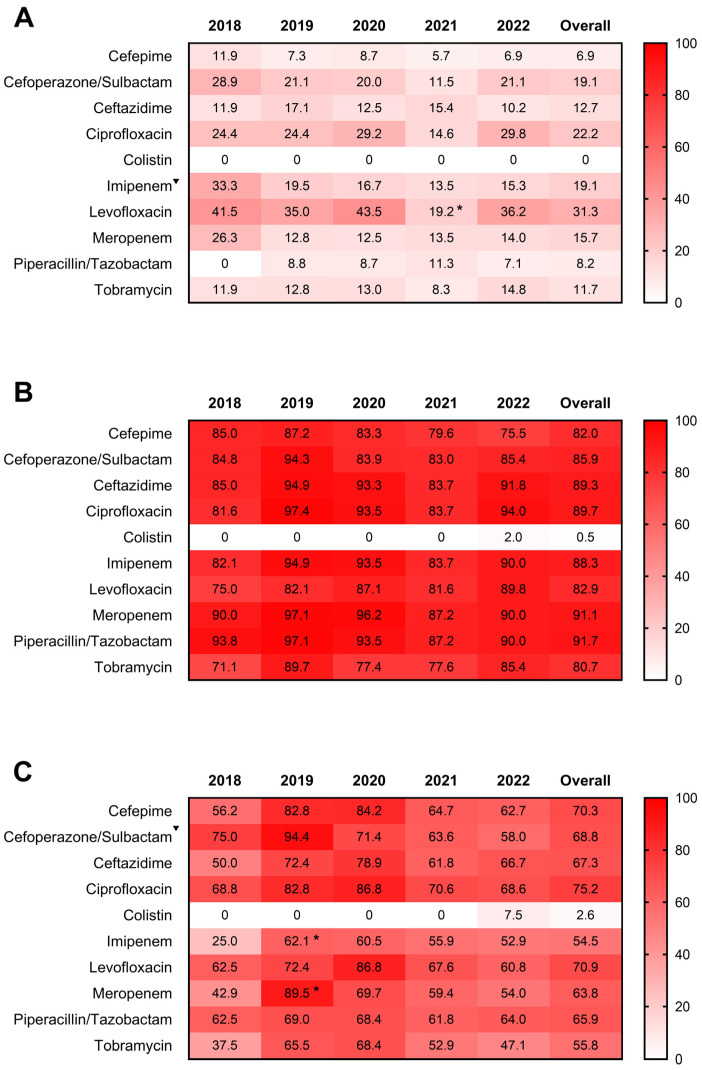
Antimicrobial resistance profiles of *P. aeruginosa* (**A**), *A. baumannii* complex (**B**), and *K. pneumoniae* (**C**) isolated from sputum samples of respiratory inpatients from 2018 to 2022. Numbers in the heat maps represented the percentage of antibiotic resistance to the tested drugs. The Total column represented the overall 5-year drug resistance rates. * indicated *p* < 0.05 when compared to the data in the previous years. ^▼^ indicated *p* < 0.05 for the drug-resistant trend from 2018 to 2022.

We further analyzed the antimicrobial resistance patterns of *P. aeruginosa*, *A. baumannii* complex, and *K. pneumoniae* from sputum samples of hospitalized respiratory patients. Overall patterns indicated that *A. baumannii* complex had the highest resistance to tested antibiotics compared with *P. aeruginosa* and *K. pneumoniae* (Figure 4). The 5-year drug resistance rate of *P. aeruginosa* to levofloxacin was the highest (31.3%), followed by ciprofloxacin (22.2%) and cefoperazone/sulbactam and imipenem ranked third (19.1%) (Figure 4A). The drug resistance rates to levofloxacin significantly decreased in 2021 (*p* = 0.046). In addition, the annual resistance rates of *P. aeruginosa* to imipenem showed a declining trend from 2018 to 2022 (*p* = 0.0239). The resistance rates to meropenem ranged from 12.5% to 26.3% over 5 years, but the difference was not statistically significant (*p* = 0.1988). Notably, *A. baumannii* complex exhibited a complete resistance profile to all tested drugs except colistin between 2018 and 2022 (Figure 4B). As for *K. pneumoniae*, the resistance rate to imipenem and meropenem significantly increased in 2019 (*p* < 0.05), while that to cefoperazone/sulbactam showed a decreasing trend during the study period (*p* = 0.0110) (Figure 4C). In addition, the resistance rates to the other tested drugs except colistin remained at high levels, and the annual change trend was not statistically significant (Figure 4C). Notably, *A. baumannii* complex and *K. pneumoniae* were susceptible to colistin from 2018 to 2021, whereas they developed resistance in 2022. In contrast, *P. aeruginosa* remained persistently susceptible to colistin over the past 5 years.

### 2.5. Prevalence of Carbapenem-Resistant Bacteria Infection

**Figure 5:**
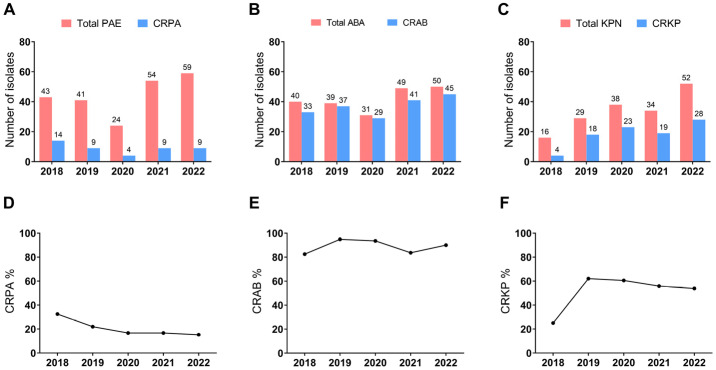
Prevalence of Carbapenem-resistant bacteria infection in sputum samples of respiratory inpatients from 2018 to 2022. (**A**) Numbers of total detected *P. aeruginosa* and CRPA. (**B**) Numbers of total detected *A. baumannii* complex and CRAB. (**C**) Numbers of total detected *K. pneumoniae* and CRKP. (**D**–**F**) The proportion of CRPA (**D**), CRAB (**E**), and CRKP (**F**) in their respective identified bacteria. PAE, *P. aeruginosa*; CRPA, Carbapenem-resistant *P. aeruginosa*; ABA, *A. baumannii* complex; CRAB, Carbapenem-resistant *A. baumannii*; KPN, *K. pneumoniae*; CRKP, Carbapenem-resistant *K. pneumoniae*.

As Carbapenem-resistant Gram-negative bacteria become major threats to global public health [16], we further analyzed the prevalence of carbapenem-resistant *P. aeruginosa* (CRPA), *A. baumannii* (CRAB), and *K. pneumoniae* (CRKP) isolates in positive sputum cultures of hospitalized respiratory patients (Figure 5). In total, the number of CRAB detected in sputum was larger than that of CRPA or CRKP. The number of CRPA and CRAB decreased in 2020 and increased in 2021 (Figure 5A,B). In contrast, the number of CRKP increased steadily year by year except for 2021 (Figure 5C).

The proportion of CRPA in the total detected *P. aeruginosa* ranged from 15.25% to 32.56% and decreased year by year (*p* = 0.0322) (Figure 5D). After reaching a peak of 94.87% in 2019, the proportion of CRAB decreased in the following two years but eventually increased to 90% in 2022 (Figure 5E). In addition, the proportion of CRKP peaked at 62.07% in 2019 but sightly declined year by year over the following years (Figure 5F), and the trend was not statistically significant (*p* = 0.4075).

## 3. Discussion

Patients in the respiratory departments usually have underlying respiratory diseases that reduce their respiratory immunity, causing them to be vulnerable to bacterial infections, especially infections caused by multidrug-resistant (MDR) pathogens [17]. Since the outbreak of COVID-19, there has been considerable concern about the widespread use and overexposure of antibiotics, which may increase the burden of antimicrobial resistance, with far-reaching consequences. A great deal of attention has been paid to preventing and controlling nosocomial infections and antimicrobial resistance at home and abroad [18]. In the current study, we compared the clinical distribution and antibiotic resistance of bacterial infection among hospitalized patients in the respiratory unit of Guangzhou First People’s Hospital before and after the COVID-19 pandemic. The aim of the current study was to provide a reference basis for establishing strategies to control antibiotic-resistant infections.

Between January 2018 and December 2022, a total of 36,829 clinical specimens, including sputum, BALF, blood, and urine, were collected from 16,073 respiratory inpatients. Most of the patients were older adults and had co-morbidities. The bacterial burden of the four types of specimens fluctuated during the epidemic period and decreased significantly in 2021, which may have been related to the effective epidemic control situation in the region. However, the positive rates of different specimens increased in 2022, which may be due to the spread of COVID-19 peaking in Guangzhou in 2022.

The distribution of bacterial species in sputum, BALF, and blood samples was basically consistent with previous findings reported by the China Antimicrobial Surveillance Network (CHINET) 2022 [19]. One interesting finding is that the frequency of urinary tract infections in respiratory patients was relatively higher when compared to respiratory or bloodstream infections. In particular, *Enterococcus faecium* was the most commonly isolated organism in urine specimens in the present study, which, however, was less common overall in other studies [20,21] and the findings reported by CHINET 2022 [19]. Urinary tract infections associated with catheters are common among elderly patients in hospital settings, which account for 30~40% of all nosocomial infections [21]. The incidence of urinary tract infections caused by *Enterococcus* spp. has substantially increased in healthcare settings and in adults with chronic indwelling catheters [22,23]. The majority of our patients were men who were 65 years of age and older and had chronic illnesses (such as hypertension and diabetes). Such patients were in poor conditions, and indwelling catheters (e.g., endotracheal, arteriovenous, and urinary tubes) were very common among them, which might be an important factor leading to the high proportion of *Enterococcus faecium* from urine specimens.

Consistent with previous reports [24,25,26], the most reported pathogens were identified in sputum samples, with *P. aeruginosa*, *A. baumannii* complex, *Candida albicans*, and *K. pneumoniae* being the most abundant microbial isolates. The difference is that *P. aeruginosa* was the most abundant bacterial species in the respiratory tract samples in our study, while *K. pneumoniae,* reported by CHINET, ranked 1st [24]. *P. aeruginosa* is an obligate aerobic bacterium that is more responsible for infection of the respiratory tract than the urinary tract and other organs [27]. In addition, *Candida* species were detected in all specimens and were abundant in respiratory tract samples. Infections caused by opportunistic *Candida* are common in patients with asthma, one of the most common respiratory diseases [28]. Asthma patients require long-term use of inhaled steroids, which can lead to immune problems in the host and cause opportunistic *Candida* infections [28]. Furthermore, the present study supports previous studies regarding the decline of *Haemophilus influenzae* in China under the impact of the COVID-19 pandemic [29,30]. *Haemophilus influenzae* is mainly transmitted through respiratory secretion droplets and direct close contact [31]. The social distance and personal protection-related measures, especially the use of masks, may have contributed to this decrease. However, the annual isolation of *A. baumannii* complex and *Streptococcus pneumoniae* did not show a declining trend as previously reported [32,33].

As the main pathogenic bacteria separated from sputum, we further analyzed the tendency of the susceptibility and resistance of *P. aeruginosa*, *A. baumannii* complex, and *K. pneumoniae* isolates. Overall, *A. baumannii* complex maintained complete resistance to all tested antibiotics except colistin over the past 5 years. The resistance rates of *K. pneumoniae* were much lower than those of *A. baumannii* complex during the study period, but they still remained at high levels. Compared with *A. baumannii* complex and *K. pneumoniae*, the resistance rates of *P. aeruginosa* were the lowest.

There were different degrees of resistance to different antibiotics. Antimicrobial resistance results showed that *P. aeruginosa* was mainly resistant to levofloxacin, ciprofloxacin, cefoperazone/sulbactam, and imipenem. Among them, the resistance rates to the first three drugs were higher than those reported by CHINET in the same year [34], indicating that the frequency and intensity of treatment of fluoroquinolones and third-generation cephalosporins for *P. aeruginosa* infection in our department have increased in the past five years. In addition, the drug resistance of *P. aeruginosa* to imipenem and meropenem was lower than that reported by CHINET in the same year [34]. Notably, *A. baumannii* complex isolated from sputum exhibited a complete resistance profile to all tested drugs except colistin and had the highest resistance compared to *P. aeruginosa* and *K. pneumoniae.* Similarly, several studies in other countries have reported that *A. baumannii* was the most resistant pathogen isolated from clinical specimens in hospitals [24,35,36,37]. These findings indicated a devastating spread of MDR *A. baumannii* worldwide, posing a severe challenge for physicians and healthcare workers. In addition, the overall resistance profiles of *K. pneumoniae* to the tested antibiotics, except colistin, still remained at high levels during the epidemic. In 2022, the resistance rates of the tested drugs other than colistin and tobramycin exceeded 50%, significantly higher than those reported by CHINET [34]. This antibiogram pattern of *K. pneumoniae* is also worrisome as few antibiotics retain activity against them, and they are hard to eliminate. Furthermore, colistin is a critical last-resort drug against MDR bacteria [38]. Our data showed the emergence of colistin-resistant *A. baumannii* complex and *K. pneumoniae* in our hospital in 2022, which requires urgent attention to identify combination therapy active towards this emerging resistance.

Carbapenem resistance in Gram-negative bacteria is on the rise globally. CRPA, CRAB, and CRKP rank the top on a list of priority bacterial pathogens by the World Health Organization (WHO) [39]. COVID-19 has affected bacterial healthcare-associated infections in many aspects, with an increase in the incidence of carbapenem-resistant organisms reported at some hospitals compared with before the pandemic [3,40], leading to stronger pathogenicity and mortality. We found a substantial prevalence of antimicrobial resistance for several WHO critical pathogens in the past five years, including CRPA (20.4% of isolates were carbapenem-resistant), CRAB (88.5% were carbapenem-resistant), and CRKP (54.4% were carbapenem-resistant). Although the proportion of CRPA and CRKP slowly declined during the COVID-19 pandemic, the number of isolated strains did not follow the decreasing trend. Notably, the number and proportion of CRAB increased in 2022 compared to those in 2021, and the upward trend cannot be ignored. The detection rates of CRPA, CRAB, and CRKP in Guangdong were 9.8%, 78.5%, and 14.8% in 2022 [19], respectively, much lower than the detection rates of this study (15.2%, 90.0%, and 53.8%, respectively). The high proportion of carbapenem-resistant Gram-negative bacteria was associated with increased antibiotic exposure and horizontal transmission of plasmids [16]. Epidemiological investigations have shown that excessive use of antibiotics promotes the reproduction and transmission of antibiotic genes in the environment [41], exacerbating the generation and spread of new MDR bacteria. Therefore, medical staff should implement a reasonable prescription drug system in clinical work, do good work in diagnosis, and minimize the use of empiric broad-spectrum antibiotics.

In conclusion, our data highlight the high prevalence of Carbapenem-resistant Gram-negative bacteria in respiratory inpatients during the COVID-19 pandemic. Although the resistance trend in a few antibiotics was slowing down, the overall trend still remained at high levels. Therefore, hospitals need to strengthen antimicrobial resistance surveillance and improve therapeutic management to contain the generation and spread of bacterial resistance.

## 4. Materials and Methods

### 4.1. Collection of Specimens for Bacterial Investigation

We retrospectively reviewed the medical records of 16,073 patients who were treated at the PCCM department of Guangzhou First People’s Hospital, Guangdong, China, from 2018 to 2022. The study hospital is one of the largest hospitals in Guangdong Province, and the PCCM department, with 87 beds, is a key clinical specialty in Guangdong Province. Various bacterial isolates derived from clinical specimens, including sputum, BALF, blood, and urine, were collected. Standard microbiological techniques were used for the isolation and identification of the causative bacteria as previously described [42]. Briefly, blood was collected in BACTEC bottles and incubated in Bactec FX (bioMérieux, Inc., Marcy-l’Étoile, France) instruments for a maximum of 5 days. Sputum, BALF, and urine specimens were plated on blood and chocolate agar plates (Crmicrobio, Jiangmen, China). For positive cultures, the IVD MALDI Biotype system (Bruker Daltonics, GmbH, and Co., Bremen, Germany) and the VITEK-2 Compact automatic microbiology system (bioMérieux) were used to identify the bacterial species. The first strain isolated from each patient was included, while the repeated strains obtained from the same case and the same site were excluded.

### 4.2. Antibiotic Susceptibility Testing

Antibiotic susceptibility testing was performed using the Vitek-2 Compact automatic microbiology system. Some drug sensitivity was supplemented by the Kirby-Bauer paper diffusion method (OXOID Limited, Basingstoke, UK). The results reported as ‘susceptible’ or ‘resistant’ were interpreted according to the guidelines of the Clinical and Laboratory Standards Institute (CLSI) [43]. *E. coli* ATCC 25,922 and *P. aeruginosa* ATCC 27853 strains were used for quality control.

### 4.3. Analysis of Demographic and Clinical Data

Demographic and clinical data of patients were obtained from the hospital information system available on the hospital intranet.

### 4.4. Statistical Analysis

WHONET 5.6 software and GraphPad Prism 8 were used to process and analyze the data of bacterial distribution and antibiotic resistance profiles. Comparison between different years of bacterial detection and drug resistance rates of pathogens were analyzed by Four-table or multiple independent sample contingency table χ^2^ (and Fisher’s exact) tests. Linear by linear χ^2^ test was further used to analyze the time trend, and results with a *p* value < 0.05 were considered statistically significant.

### 4.5. Ethics Statement

The protocol was approved by the Ethical Committee of Guangzhou First People’s Hospital. An informed consent was not required because this was a retrospective study with no interaction with patients. Patient privacy and confidentiality of data were maintained in accordance with The Declaration of Helsinki.

## Figures and Tables

**Table 1:** The demographic and clinical characteristics of respiratory inpatients from 2018 to 2022.

	2018	2019	2020	2021	2022	Total
**Gender**						
Male	2201	2228	1776	2189	2097	10,491
Female	1244	1298	934	1074	1032	5582
**Age**						
<15	7	15	4	3	3	32
15–24	116	101	75	70	50	412
25–34	127	162	81	92	92	554
35–44	168	180	118	128	113	707
45–54	319	315	294	351	332	1611
55–64	743	769	572	704	603	3391
>64	1965	1984	1566	1915	1936	9366
**Co-morbidities**						
Hypertension	1466	1590	1137	1316	1306	6815
Diabetes	698	639	546	579	582	3044
COPD	776	811	551	764	693	3595
Asthma	150	172	115	94	152	683

**Table 2:** The pathogen distribution in sputum cultures of respiratory inpatients from 2018 to 2022.

Pathogen	2018 (n = 233)	2019 (n = 254)	2020 (n = 271)	2021 (n = 299)	2022 (n = 357)
*Pseudomonas aeruginosa*	43 (18.45%)	41 (16.14%)	24 (8.86%) *	54 (18.06%)	59 (16.53%)
*Acinetobacter baumannii* complex	40 (17.17%)	39 (15.35%)	31 (11.44%)	49 (16.39%)	50 (14.01%)
*Candida albicans*	41 (17.60%)	25 (9.84%) *	29 (10.70%)	38 (12.71%)	51 (14.29%)
*Klebsiella pneumoniae*	16 (6.87%)	29 (11.42%)	38 (14.02%) *	34 (11.37%)	52 (14.57%)
*Stenotrophomonas maltophilia*	9 (3.86%)	9 (3.54%)	21 (7.75%) *	28 (9.36%)	21 (5.88%)
*Haemophilus influenzae*	23 (9.87%)	35 (13.78%)	11 (4.06%) ***	8 (2.68%)	8 (2.24%)
*Streptococcus pneumoniae*	20 (8.58%)	11 (4.33%)	11 (4.06%) *	17 (5.69%)	14 (3.92%)
*Staphylococcus aureus*	8 (3.43%)	7 (2.76%)	4 (1.48%)	11 (3.68%)	32 (8.96%)
*Burkholderia cepacia* complex	13 (5.58%)	6 (2.36%)	22 (8.12%)	5 (1.67%)	6 (1.68%)
*Moraxella catarrhalis*	8 (3.43%)	12 (4.72%)	4 (1.48%)	11 (3.68%)	7 (1.96%)
*Escherichia coli*	4 (1.72%)	8 (3.15%)	5 (1.84%)	6 (2.01%)	9 (2.52%)
Other pathogens	8 (3.43%)	32 (12.60%)	71 (26.20%)	38 (12.71%)	48 (13.45%)

* indicated *p* < 0.05, *** indicated *p* < 0.001, when compared to the data in the previous years.

## Data Availability

The data used to support the findings of this study are available from the corresponding author upon request.

## Supplementary Materials

The following supporting information can be downloaded at https://www.mdpi.com/article/10.3390/microorganisms11102542/s1, Table S1: Overall distribution of Pathogens in sputumn samples; Table S2: Overall distribution of Pathogens in BALF samples; Table S3: Overall distribution of Pathogens in blood samples; Table S4: Overall distribution of Pathogens in urine samples.
